# Exercise Training Attenuates Acute β-Adrenergic Receptor Activation-Induced Cardiac Inflammation via the Activation of AMP-Activated Protein Kinase

**DOI:** 10.3390/ijms24119263

**Published:** 2023-05-25

**Authors:** Mi Zhang, Akehu Alemasi, Mingming Zhao, Wenli Xu, Youyi Zhang, Wei Gao, Haiyi Yu, Han Xiao

**Affiliations:** 1 Department of Cardiology and Institute of Vascular Medicine, Peking University Third Hospital, Beijing 100191, China; 2 State Key Laboratory of Vascular Homeostasis and Remodeling, Peking University, Beijing 100191, China; 3 NHC Key Laboratory of Cardiovascular Molecular Biology and Regulatory Peptides, Beijing 100191, China; 4 Beijing Key Laboratory of Cardiovascular Receptors Research, Beijing 100191, China; 5 Research Unit of Medical Science Research Management/Basic and Clinical Research of Metabolic Cardiovascular Diseases, Chinese Academy of Medical Sciences, Beijing 100191, China

**Keywords:** acute sympathetic stress, exercise training, NLR family, pyrin domain-containing 3, AMP-activated protein kinase

## Abstract

Exercise has proven cardiac benefits, but the underlying mechanisms of exercise that protect the heart from acute sympathetic stress injuries remain unknown. In this study, adult C57BL/6J mice and their AMP-activated protein kinase α2 knockout (AMPKα2^−/−^) littermates were either subjected to 6 weeks of exercise training or housed under sedentary conditions and then treated with or without a single subcutaneous injection of the β-adrenergic receptor (β-AR) agonist isoprenaline (ISO). We investigated the differences in the protective effects of exercise training on ISO-induced cardiac inflammation in wild-type (WT) and AMPKα2^−/−^ mice using histology, enzyme-linked immunosorbent assay (ELISA) and Western blotting analyses. The results indicated that exercise training alleviated ISO-induced cardiac macrophage infiltration, chemokines and the expression of proinflammatory cytokines in wild-type mice. A mechanism study showed that exercise training attenuated the ISO-induced production of reactive oxygen species (ROS) and the activation of NLR Family, pyrin domain-containing 3 (NLRP3) inflammasomes. In cardiomyocytes, the ISO-induced effects on these processes were inhibited by AMP-activated protein kinase (AMPK) activator (metformin) pretreatment and reversed by the AMPK inhibitor (compound C). AMPKα2^−/−^ mice showed more extensive cardiac inflammation following ISO exposure than their wild-type littermates. These results indicated that exercise training could attenuate ISO-induced cardiac inflammation by inhibiting the ROS-NLRP3 inflammasome pathway in an AMPK-dependent manner. Our findings suggested the identification of a novel mechanism for the cardioprotective effects of exercise.

## 1. Introduction

Cardiovascular diseases (CVDs) are still the number one risk factor threatening human life and health [1]. Acute sympathetic stress resulting in β-adrenergic receptor (β-AR) over-activation is an important pathological factor in cardiac diseases [2]. It is well known that acute sympathetic stress is usually unpredictable [3,4] and the current treatment method following cardiac injuries is symptomatic support treatment as cardiac pathological remodeling, which is difficult to reverse, once it has already occurred [5,6]. Therefore, we urgently need an effective way to prevent cardiac injuries that are caused by acute sympathetic stress.

An increasing body of evidence has demonstrated that exercise training can improve status and outcomes in patients with existing cardiovascular diseases [7,8] and reduce the risks of the occurrence of cardiovascular diseases and cardiovascular events [9]. Epidemiological studies have demonstrated that higher numbers of steps per day are associated with reduced CVD risk, incidence and mortality [10]. The results of meta-analyses and systematic reviews have also shown that engaging in physical activity leads to a reduced risk of cardiovascular disease mortality [11], a reduced all-cause hospitalization rate for patients with heart failure (HF) and a reduced hospitalization rate for HF [12]. Acute sympathetic stress can trigger and aggravate cardiac diseases. The main pathological basis of acute sympathetic stress-induced cardiac injuries is cardiac remodeling, including cardiac inflammatory response, cardiac fibrosis and cardiomyocyte apoptosis. Inflammation is a common pathological driver for the development and progression of CVDs [13]. Several studies have found that exercise can suppress cardiac inflammation; for example, exercise training has been shown to improve cardiac inflammation in aging patients [14], as well as chronic heart failure [15]. However, it remains unclear as to whether and how exercise training has protective effects against sympathetic stress-induced cardiac inflammation.

In our previous study, we found that acute sympathetic stress activated β-AR and induced NLR Family, pyrin domain-containing 3 (NLRP3) inflammasome activation and IL-18 cleavage, which, in turn, led to the release of chemokines and pro-inflammatory cytokines in cardiac tissue, resulting in cardiac dysfunction [16]. Numerous studies have confirmed that the inflammatory response can aggravate tissue necrosis and apoptosis in myocardial pathologic injuries and that NLRP3 inflammasomes play an important role in this process [17,18,19]. It is unknown whether exercise can inhibit the cardiac NLRP3 inflammasome activation that is induced by acute sympathetic stress.

The occurrence and development of inflammation are closely related to cell energy metabolism. The AMP-activated protein kinase (AMPK) is a key kinase in maintaining cell energy homeostasis, and research has shown that AMPK has an extremely important regulatory function in the inflammatory response [20]. Our previous findings showed that 4 weeks of swimming training alleviated isoprenaline (ISO)-induced cardiac fibrosis and oxidative stress in WT mice, but not in AMPKα2^−/−^ mice [21]. Therefore, we proposed a study on whether exercise training could alleviate acute sympathetic stress-induced cardiac inflammation by activating AMPK to inhibit the excessive activation of NLRP3 inflammasomes in the heart.

Thus, in our present study, we investigated the protective effects of exercise training against β-AR overactivation-induced cardiac inflammation. We also identified the underlying in vivo and in vitro mechanisms by which exercise training inhibited β-AR overactivation-induced cardiac NLRP3 inflammasome activation and inflammation in an AMPK-dependent manner.

## 2. Results

### 2.1. Exercise Training Attenuated Isoprenaline-Induced Cardiac Inflammation

**Figure 1:**
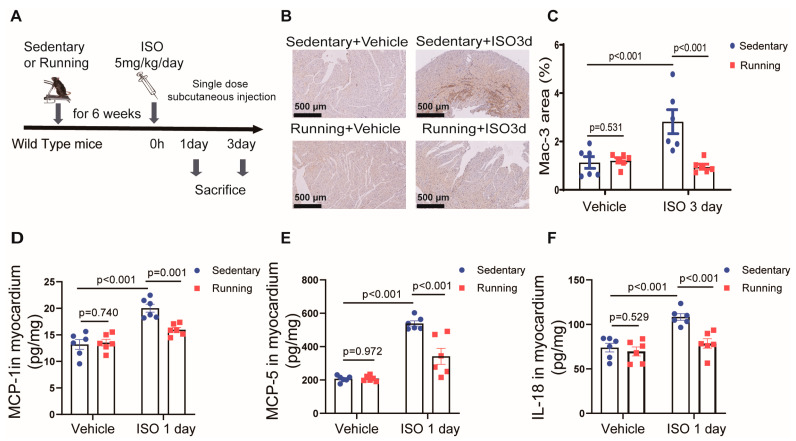
Exercise training could inhibit ISO-induced cardiac inflammation: (**A**) a pattern diagram of the experimental exercise training protocol; (**B**,**C**) representative immunostainings and quantifications of Mac-3 (macrophage marker) in the hearts on the third day after ISO treatment (bar = 500 μm); (**D**–**F**) the concentrations of MCP-1 (**D**), MCP-5 (**E**) and IL-18 (**F**) in the myocardium of the mice, which were determined using ELISA kits. Note: ISO, isoprenaline; WT, wild-type; Running, exercise training; MCP-1, monocyte chemoattractant protein-1; MCP-5, monocyte chemoattractant protein-5; IL-18, interleukin-18; *n* = 6. The data are the mean ± SEM from a one-way ANOVA with a Tukey’s post-hoc test.

We initially tested whether exercise training could attenuate the cardiac inflammation induced by ISO insult. We created a working model of the animal exercise training protocol, experimental strategy and schedule (Figure 1A). The macrophage marker Mac-3-positive areas in the mouse hearts significantly increased on the third day following the ISO injection, but pretreatment with exercise training could significantly reduce these increases, suggesting that exercise training could reduce ISO-induced macrophage infiltration in mouse hearts (Figure 1B,C). At the same time, we performed cardiac ultrasound examinations on the WT mice 3 days after ISO stimulation and found that cardiac diastolic and systolic functions were not altered in the ISO group (Figure S1A–C). We also found that hypertrophy and fibrosis did not occur in the hearts of mice 3 days after ISO stimulation (Figure S2A,B,E,F). Our previous study found increases in chemokines (monocyte chemoattractant protein-1 (MCP-1) and monocyte chemoattractant protein-1 (MCP-5)) during ISO-induced cardiac inflammation, which were induced by the cleavage-activated interleukin-18 (IL-18), but not interleukin-1β (IL-1β), and these chemokines, in turn, contributed to macrophage infiltration and cardiac inflammatory responses [16]. On the first day following the ISO injection, the mice in the sedentary group had significantly higher levels of inflammatory markers, such as MCP-1 (Figure 1D), MCP-5 (Figure 1E) and IL-18 (Figure 1F), in their cardiac tissue; in contrast, the mice in the exercise training group had lower levels of these inflammatory factors. Moreover, no significant changes of IL-1β contents were observed in the heart at 1 or 3 days after ISO treatment (Figure S3A).

### 2.2. Exercise Training Alleviated Isoprenaline (ISO)-Induced Reactive Oxygen Species (ROS) Production and Inflammasome Activation

**Figure 2:**
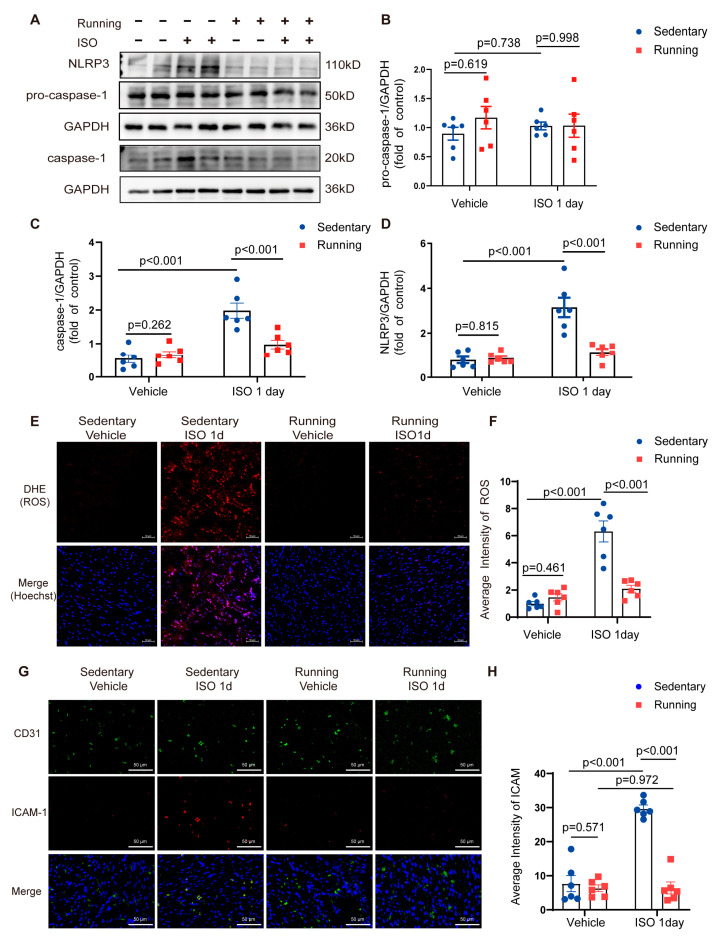
Exercise training inhibited the ISO-induced activation of NLRP3 inflammasomes and increases in ROS and ICAM-1 production: (**A**) the Western blot analysis of pro-caspase-1, caspase-1 and NLRP3 levels in WT mice heart tissue; (**B**–**D**) the quantitative analysis of the relative protein expression of (**B**) pro-caspase-1, (**C**) caspase-1 and (**D**) NLRP3 in the mouse heart tissue; (**E**,**F**) (**E**) the fluorescent microscopy of representative DHE staining for ROS levels in the left ventricle (LV) sections from wild-type mice (bar = 50 μm) and (**F**) the quantification of the fluorescence intensity of DHE in the heart sections; (**G**,**H**) (**G**) the fluorescent microscopy of representative staining for ICAM-1 levels in the left ventricle (LV) sections from wild-type mice (bar = 50 μm) and (**H**) the quantification of the fluorescence intensity of ICAM-1 in the heart sections. Note: ISO, isoprenaline; Running, exercise training; WT, wild-type; LV, left ventricle; CD31, vascular endothelial cell maker; ICAM-1, intercellular adhesion molecule 1; *n* = 6. The data are the mean ± SEM from a one-way ANOVA with a Tukey’s post-hoc test.

In the sedentary mice group, inflammasomes were activated, as evidenced by the increases in the caspase-1 and NLRP3 protein levels in the mouse hearts on the first day following ISO insult (Figure 2A,C,D), whereas the pro-caspase-1 levels showed no change (Figure 2B). The levels of ROS (Figure 2E,F) and intercellular adhesion molecule 1 (ICAM-1) (Figure 2G,H) increased on the first day after ISO insult, which was consistent with the preceding changes in inflammatory activation. Compared to the sedentary group, the exercise-training mice had reduced ROS and adhesion molecule (ICAM-1) production and inflammasome activation levels. These findings showed that exercise training prevented the ISO-induced activation of cardiac inflammasomes.

### 2.3. AMP-Activated Protein Kinase (AMPK) Activation Alleviated Isoprenaline-Induced Inflammasome Activation in Cardiomyocytes

**Figure 3:**
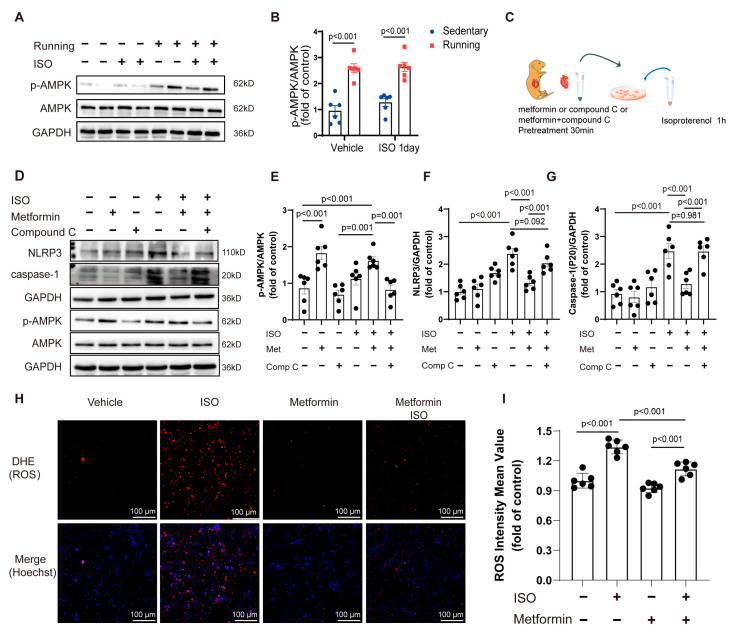
Exercise could activate AMPK, and AMPK activation could inhibit ISO-induced ROS production and NLRP3 inflammasome activation in NMCMs: (**A**,**B**) (**A**) the Western blot analysis of the protein levels of phosphorylated and total AMPK in wild-type mice under sedentary or exercise training conditions and (**B**) the quantification of phosphorylated AMPK relative to total AMPK; (**C**) a pattern diagram of the experimental protocol for the NMCMs (the NMCMs were pretreated with an AMPK activator (1 mM of metformin), AMPK inhibitor (0.1 μM of compound C) or AMPK activator (metformin) + AMPK inhibitor (compound C) for 30 min and then treated with ISO (10 μM) for 1 h); (**D**–**G**) (**D**) the protein levels of p-AMPK, NLRP3 and caspase-1 (p20) at 1 h after ISO (10 μM) treatment in mouse cardiomyocytes that were pretreated with the AMPK activator (1 mM of metformin) or AMPK inhibitor (0.1 μM of compound C) for 30 min and the quantitative analysis of the relative protein expression of (**E**) p-AMPK, (**F**) NLRP3 and (**G**) caspase-1 in the NMCMs; (**H**,**I**) the average ROS signal intensity in the NMCMs, which was measured after DHE staining (bar = 100 μm). Note: ISO, isoprenaline; Met, metformin; Comp C, compound C; Running, exercise training; *n* = 6. The data are the mean ± SEM from a one-way ANOVA with a Tukey’s post-hoc test or a Kruskal–Wallis ANOVA with a post-hoc Dunn’s multiple comparison test.

Engaging in exercise training for 6 weeks significantly increased the level of AMPK phosphorylation compared to that in animals under sedentary conditions, showing the effects of AMPK activation (Figure 3A,B). We measured the heart rate and blood pressure of the mice and found no changes in heart rate or blood pressure at 1 or 3 days after ISO insult (Figure S4), while mice in the exercise group showed a decrease in heart rate and no variation in blood pressure. The plasma levels of catecholamines were also determined and we found that ISO stimulation increased catecholamines (Figure S5); however, exercise was able to downregulate the levels of catecholamines. Exercise training with ISO treatment also enhanced AMPK phosphorylation compared to that in the sedentary group. We used an AMPK activator (metformin) to mimic the process of AMPK activation at the cellular level and demonstrate that the activation of AMPK could be an important reason for the reduction in myocardial inflammation after exercise training. Before stimulating cardiomyocytes with ISO (10 μM), we pretreated the cardiomyocytes with the AMPK activator (metformin, 1 mM) or AMPK inhibitor (compound C, 0.1 μM) for 30 min. In addition, in order to verify that the effect of the metformin was AMPK-dependent, we used metformin and compound C to treat cells at the same time to inhibit metformin-induced AMPK activation (Figure 3C). Our results showed that AMPK activator pretreatment could reduce the level of ISO-induced NLRP3 and caspase-1 (P20) inflammasome activation in cardiomyocytes. In contrast, these effects of the AMPK activator were reversed by the AMPK inhibitor (Figure 3D–G). Additionally, we found that pretreatment with the AMPK activator prevented ISO-induced ROS production (Figure 3H,I). Based on these results, the ISO-induced inflammasome activation in cardiomyocytes appeared to be inhibited by AMPK activation.

### 2.4. Exercise Training Failed to Reduce Isoprenaline-Induced Cardiac Inflammation in AMPKα2^−/−^ Mice

**Figure 4:**
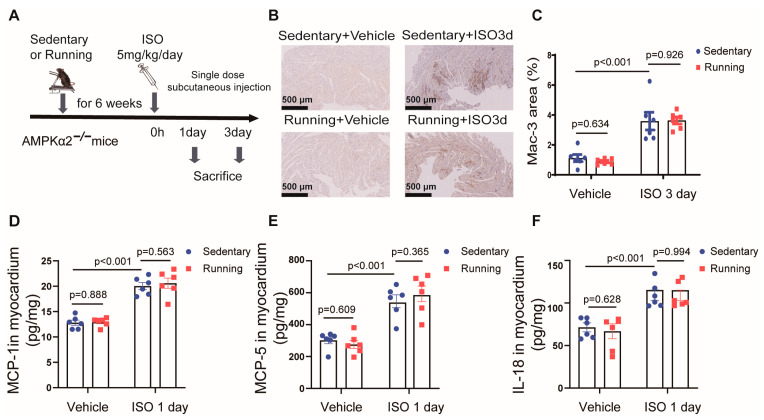
AMPKα2^−/−^ mice failed to reduce ISO-induced cardiac inflammation: (**A**) the protocol that was used for the evaluation of the effects of exercise training on ISO-induced cardiac inflammation in AMPKα2^−/−^ mice; (**B**,**C**) Mac-3 (macrophage marker) staining, which was used to calculate the area of macrophage infiltration (compared to the sedentary group, the area of macrophage infiltration did not decrease in AMPKα2^−/−^ mice after exercise training (bar = 500 μm)); (**D**–**F**) the concentrations of (**D**) MCP-1, (**E**) MCP-5 and (**F**) IL-18 in the myocardium of the AMPKα2^−/−^ mice, which was determined using an ELISA. Note: ISO, isoprenaline; Running, exercise training; MCP-1, monocyte chemoattractant protein-1; MCP-5, monocyte chemoattractant protein-5; IL-18, interleukin-18; *n* = 6. The data are the mean ± SEM from a one-way ANOVA with a Tukey’s post-hoc test.

We then investigated whether AMPK was critical for the inhibitory effect of exercise training on ISO-induced cardiac inflammation. AMPKα2^−/−^ mice underwent identical procedures to those presented in Figure 1A, including exercise training or sedentary activity with or without ISO administration (Figure 4A). Macrophage infiltration was enhanced in the hearts of AMPKα2^−/−^ animals in the exercise training group on day 3 after ISO treatment, as demonstrated by the immunohistochemistry labeling of the macrophage marker Mac-3 (Figure 4B,C). Similarly, we performed a cardiac ultrasonography examination on the AMPKα2^−/−^ mice 3 days after ISO stimulation and found that there were no significant changes in the cardiac diastolic and systolic function of the mice (Figure S1D–F). We also found that hypertrophy and fibrosis did not occur in the hearts of mice 3 days after ISO stimulation (Figure S2C,D,G,H). Hence, exercise training did not reduce ISO-induced macrophage infiltration in AMPKα2^−/−^ mice. Similarly, exercise training did not decrease the elevated levels of chemokine factor MCP-1 (Figure 4D), MCP-5 (Figure 4E) or proinflammatory cytokine IL-18 (Figure 4F) induced by ISO treatment in AMPKα2^−/−^ mice.

### 2.5. Exercise Training Failed to Reduce the Isoprenaline-Induced Activation of Cardiac NLRP3 Inflammasomes or the Production of Reactive Oxygen Species (ROS) and ICAM-1 in AMPKα2^−/−^ Mice

**Figure 5:**
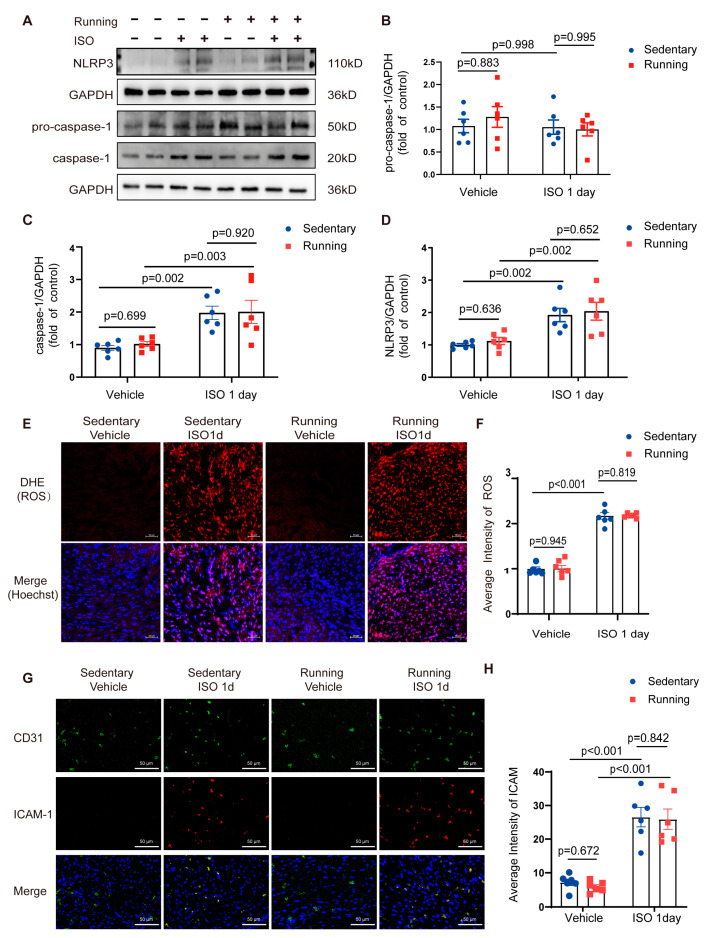
Exercise training reduced ISO-induced cardiac inflammation in an AMPK-dependent manner: (**A**–**D**) heart tissue was collected and Western blotting was used to detect and quantitatively analyze the relative protein expression of (**B**) pro-caspase-1, (**C**) caspase-1 and (**D**) NLRP3 in the heart tissue; (**E**,**F**) (**E**) the fluorescent microscopy of representative DHE staining for ROS levels in the left ventricle (LV) sections from AMPKα2^−/−^ mice (bar = 50 μm) and (**F**) the quantification of the fluorescence intensity of DHE in the AMPKα2^−/−^ sections (*n* = 6); (**G**,**H**) (**G**) the fluorescent microscopy of representative staining for ICAM-1 levels in the left ventricle (LV) sections from AMPKα2^−/−^ mice (bar = 50 μm) and (**H**) the quantification of the fluorescence intensity of ICAM-1 in the heart sections. Note: ISO, isoprenaline; Running, exercise training; WT, wild-type; LV, left ventricle; CD31, vascular endothelial cell maker; ICAM-1, intercellular adhesion molecule 1; *n* = 6. The data are the mean ± SEM from a one-way ANOVA with a Tukey’s post-hoc test.

With exercise training, ISO treatment increased inflammasome proteins, such as caspase-1 and NLRP3, in AMPKα2^−/−^ animal hearts, and these increases were similar to those observed in the sedentary group (Figure 5A–D). ISO treatment resulted in increased levels of ROS (Figure 5E,F) and ICAM-1 (Figure 5G,H) in the exercise training AMPKα2^−/−^ mice group, which were similar to those observed in the sedentary AMPKα2^−/−^ mice group. We simultaneously compared relevant inflammatory indicators in the AMPKα2^−/−^ and WT mice and found that ISO-induced inflammation was attenuated in the WT mice following exercise but not in the AMPKα2^−/−^ mice. We also analyzed the results of the inflammatory indicators, Mac-3 staining and staining for ROS and ICAM-1 in the WT and AMPKα2^−/−^ mice and found that in the WT mice, exercise training was able to alleviate ISO-induced cardiac inflammation, while there was no such effect in the AMPKα2^−/−^ mice (Figure S6). Thus, the beneficial effects of exercise training on cardiac inflammation depended on AMPK.

## 3. Discussion

**Figure 6:**
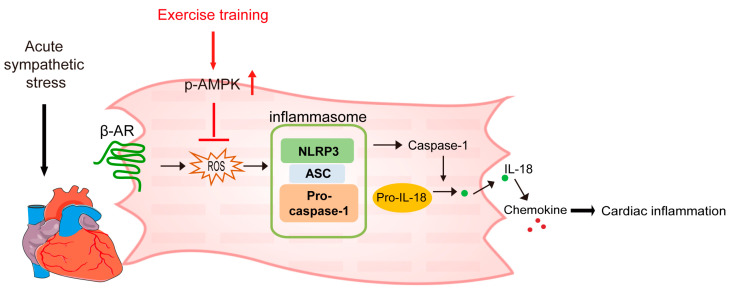
The working model showing how exercise training alleviated ISO-induced cardiac inflammation by activating AMPK. Note: β-AR, β-adrenergic receptor; p-AMPK, phosphorylated AMP-activated protein kinase; ROS, reactive oxygen species; NLRP3, NLR Family, pyrin domain-containing 3; ASC, apoptosis-associated speck-like protein containing a CARD (caspase activation and recruitment domain); IL-18, interleukin-18.

The present study demonstrated that exercise training attenuated ISO-induced cardiac inflammation in mice by reducing ROS production and NLRP3 inflammasome activation. The underlying mechanism of these effects was that exercise training activated AMPK, which directly inhibited the ROS–NLRP3 signaling pathway and attenuated acute sympathetic stress-induced cardiac inflammation (Figure 6).

The occurrence and development of heart diseases are often accompanied by the overactivation of the sympathetic system. Previous studies in this area have shown that acute exercise can stimulate sympathetic nervous system (SNS) activation and cause an acute increase in catecholamine levels, possibly leading to cardiac injuries, whilst long-term exercise can lessen sympathetic tension [22,23,24]. Chronic exercise promotes sympathetic inhibition, and regular physical activity can mitigate central and peripheral sympathetic activity, thereby plausibly reducing the risk of heart injuries, which could explain the clinical benefits of exercise for CVDs [23,25,26]. Consistent with clinical evidence, in this study, we found that cardiac inflammation resulting from β-AR overactivation was significantly ameliorated after 6 weeks of exercise training. This evidence further suggests that chronic aerobic exercise not only improves cardiac function by reducing catecholamines but also reduces the risk of cardiac injuries by antagonizing the signaling pathways downstream of the β-ARs.

A previous study found that acute mental stress resulted in inflammatory leukocyte recruitment, which contributed to the occurrence of cardiovascular events [27]. Acute sympathetic stress-induced cardiac injuries are often accompanied by inflammatory responses, such as inflammatory cell infiltration and inflammatory cytokine/chemokine release [16]. Thus, inhibiting the overactivation of the inflammatory response has been proposed as a promising strategy to attenuate pathological cardiac remodeling. The anti-inflammatory effects of exercise have been reported in a number of studies. For example, exercise training reduces inflammation in adipose tissue by inhibiting macrophage infiltration and the shift from the M1 to M2 macrophage phenotype in mice with high-fat diet-induced obesity [28], and clinical evidence has suggested that exercise training inhibits the childhood obesity-induced activation of inflammatory signaling pathways via microbiota modulation [29]. Other research has also confirmed that regular aerobic exercise can activate the PDGF-BB/PDGFR-β/PI3K/Akt/eNOS signaling pathway, which induces cardioprotection by counteracting the obesity-associated inflammatory response and dyslipidemia [30]. In our study, we found that pretreatment involving exercise training for 6 weeks could inhibit ISO-induced cardiac inflammation. The underlying mechanism of this effect was that exercise training could inhibit the production of ROS in the heart tissue, thereby inhibiting the overactivation of NLRP3 inflammasomes.

NLRP3 inflammasomes are critical in ISO-induced cardiac inflammation as they promote the cleavage and activation of IL-18, resulting in the initiation of cardiac inflammation [16]. NLRP3 activation induces a large number of pro-inflammatory cytokines, such as IL-1β and IL-18, which are involved in the development of various diseases [31]. However, there were no significant changes in IL-1β in the heart during acute β-receptor over-activation (Figure S3), which was consistent with our previous finding [16]. Our study proved that exercise training could suppress the activation of NLRP3 inflammasomes in the heart, thereby ameliorating cardiac inflammation induced by the overactivation of β-ARs. By further exploring how exercise could inhibit the activation of NLRP3 inflammasomes in the heart caused by acute sympathetic stress, we found that AMPK had an essential role in inhibiting cardiac inflammation.

AMPK, a heterotrimeric complex, is composed of a catalytic α-subunit and beta (β) and gamma (γ) regulatory subunits. The α-subunit has two isoforms, α1 and α2, encoded by gene PRKAA1 and PRKAA2, respectively [32]. Both α1 and α2 subunits were present in the heart tissue, with the α2 subunit predominating in cardiomyocytes [33]. Therefore, the KO mice we used were AMPK α2 subunit knockout mice; thus, the expression of the AMPK α1 subunit was not affected. In our Western blot experiments, we used an antibody for AMPKα, and AMPKα2^−/−^ mice still showed extremely weak bands, which was possibly due to the detection of the α1 subunit in the heart tissue (Figure S6A).

It is widely accepted that AMPK activation plays a protective role in a range of CVDs [34,35]. AMPK is activated by energy stress and/or cellular stress in response to increased adenosine triphosphate (ATP) consumption (e.g., exercise, cell proliferation, etc.) or decreased ATP production (e.g., oxidative stress, hypoxia, etc.) [36,37]. During exercise, the heart rate, catecholamine levels and cardiac contractility increase, leading to an increase in cardiac ATP consumption, which activates AMPK in the heart [38]. However, after long-term exercise, cardiac sympathetic tone and heart rate both decrease [24]. In our experiments, 6 weeks of exercise training caused decreases in heart rate and catecholamine levels in both wild-type and AMPKα2^−/−^ mice. Thus, the changes in AMPK activity after chronic exercise may be attributable to multiple mechanisms. After long-term exercise, the heart experiences physiological hypertrophy and increased contractility, leading to increased cardiac ATP consumption, increased free adenosine monophosphate (AMP) concentrations and reduced phosphocreatine concentrations in the heart, resulting in an increased AMP to ATP ratio, which, in turn, activates AMPK [38]. Several studies have reported that exercise can cause a decrease in glycogen levels in cardiac tissue, leading to AMPK activation [39,40,41]. Thus, exercise-induced AMPK activation is attributed to multiple mechanisms, involving not only heart rate and catecholamines but also energy metabolism and gluconeogenesis [38].

Exercise-induced adaptations in the heart can promote health and prevent diseases, and AMPK activation could play a critical role in conferring these benefits [42]. A previous study reported that the activation of AMPK could effectively inhibit myocardial ischemia-reperfusion injury-induced cardiac inflammation [43] and determined that exercise could enhance AMPK in aging rat cardiac cells and attenuate the activation of pro-inflammatory mediators, thus improving cardiac function [44]. Our previous study found that swimming training did not improve the production of ISO-induced ROS or cardiac fibrosis in AMPKα2^−/−^ mice [21]. In our study, we found that the activation of AMPK inhibited ISO-induced ROS production and NLRP3 inflammasome activation in the heart, both in vivo and in vitro. We also found that in both the WT mice and the AMPKα2^−/−^ mice, exercise training could significantly reduce the plasma levels of catecholamines (Figure S5). Additionally, exercise training failed to inhibit ISO-induced cardiac NLRP3 inflammasome activation and inflammation in the AMPKα2^−/−^ mice (Figure 4 and Figure 5). We believe that the mechanisms underlying the beneficial effects of exercise include reduced catecholamines (ligand) levels and attenuated downstream β-AR signaling in an AMPK-dependent manner. Our results showed that the inhibition of NLRP3 inflammasomes contributed to the protective role of exercise training against cardiac inflammation. Finally, we demonstrated that exercise training reduced NLRP3 inflammasomes via AMPK activation.

Exercise training can improve cardiac inflammation through a variety of pathways. For example, in rats with hypertension combined with ovariectomy, running training can effectively improve cardiac inflammation by reducing angiotensin II type I receptors [45]. Swimming training can also improve aging-induced cardiac inflammation by inhibiting the NF-κB pathway and suppressing the expression of COX2 and iNOS [14], while long-term swimming exercise can activate AMPK, leading to an increase in the expression of SIRT1 and PGC-1α in the cardiomyocytes of aging rats, thereby promoting cardiac metabolic adaptation and improving cardiac inflammation [44]. Exercise counters systemic and cardiac insulin resistance, as well as eNOS dysfunction caused by high fructose feeding, and effectively prevents the activation of cardiac pro-inflammatory signals [46]. During the early stages of myocardial infarction (e.g., one day after infarction), exercise can ameliorate the pathological inflammatory response in the infarcted area by inhibiting pro-inflammatory cell infiltration and accelerating the conversion of macrophages from M1 to M2 [47]. In our study, we showed that exercise training could also alleviate sympathetic stress-induced cardiac inflammation through the AMPK-ROS pathway, and this study provides a new mechanism for exercise training to ameliorate cardiac inflammation.

According to the World Health Organization (WHO), long-term moderate-intensity aerobic exercise (>150 min per week of moderate-intensity physical activity) is of great significance in preventing the development of cardiovascular diseases and improving prognoses [48]. In this study, exercise was used to maintain a 65–75% maximum oxygen consumption in mice during running, which was equivalent to moderate-intensity aerobic exercise in humans. Our results provide an elaboration of a new mechanism for the protective effect of moderate-intensity exercise on cardiovascular disease, that is, exercise can ameliorate cardiac injury caused by sympathetic stress through the AMPK-ROS pathway. Our previous study consistently found no cardiac fibrosis but inflammatory infiltration in mice 3 days after ISO stimulation, whereas, 7 days after ISO stimulation, mice showed cardiac fibrosis and decreased diastolic function [16]. Inflammatory infiltration at 3 days after ISO stimulation led to cardiac fibrosis and diastolic dysfunction at 7 days after ISO stimulation. We believe that running attenuates inflammatory infiltration in mice 3 days after ISO stimulation and then subsequently attenuates cardiac fibrosis and diastolic dysfunction. Therefore, moderate-intensity exercise can prevent sympathetic stimulation-induced cardiac fibrosis through the inhibition of cardiac ROS and NLRP3 inflammasomes at the early stage of sympathetic stress. For people who do not benefit from exercise, AMPK activator can be used to block early cardiac inflammation (e.g., by directly inhibiting ROS production), thereby preventing the progression of cardiac fibrosis and ultimately improving cardiac function. In the future, clinical research is needed to prove that long-term moderate-intensity exercise training performed in advance can prevent cardiac injury in people suffering from acute sympathetic stress.

In order to investigate the effect of acute sympathetic nerve stimulation on inflammatory damage to the heart, we injected either ISO or saline in both the sedentary and running groups; thus, we minimized the effects of the injections on the different outcomes in the two groups in our study. However, there was a limitation whereby in our experiments, the impact of the injections themselves on sympathetic and hormonal changes was not taken into consideration. Therefore, in further experiments, we should add a blank control group (no injections) to clarify whether the behavior of the injection is involved in cardiac injury induced by acute sympathetic stimulation.

## 4. Materials and Methods

### 4.1. Antibodies and Reagents

The macrophage-3 (Mac-3) antibody was purchased from BD Biosciences (San Jose, CA, USA). The NLRP3, p-AMPK, AMPK and glyceraldehyde-3-phosphate dehydrogenase (GAPDH) antibodies were purchased from Cell Signaling Technology (Danvers, MA, USA). The interleukin-18 (IL-18) enzyme-linked immunosorbent assay (ELISA) kits were purchased from MBL (code 7652, Sakae, Japan). The monocyte chemoattractant protein-1 (MCP-1) and monocyte chemoattractant protein-5 (MCP-5) ELISA kits were purchased from R&D Systems Incorporated (Minneapolis, MN, USA). The ISO (I5627), metformin (D150959) and caspase-1 (P20) antibodies were purchased from Sigma (Sigma-Aldrich, St. Louis, MO, USA).

### 4.2. Animal and Exercise Model

This study was conducted in accordance with the Use of Laboratory Animals, published by the US National Institutes of Health (NIH Publication No. 85-23, revised 2011), and the guidelines of the Peking University Health Science Center. The homozygous AMPKα2^−/−^ mice from the C57BL/6J background were kindly provided by Dr. Benoit Viollet (Institute National de la Santé et de la Recherche Médicale U567, Paris, France). The male AMPKα2^−/−^ and wild-type (WT) mice were bred in a specific, pathogen-free environment (temperature: 20–24 °C; relative humidity: 30–70%) under a 12:12 h light:dark cycle and received standard rodent food.

Then, 10-week-old WT (*n* = 36) and AMPKα2^−/−^ (*n* = 36) mice were randomly divided into exercise training and sedentary groups. The mice in the exercise training group followed a running exercise plan that involved running on a treadmill for 90 min/day and 6 days/week for 6 weeks at a velocity of 15 cm/s. At this velocity, the mice reached 80% of their maximal oxygen consumption (VO_2_max), according to exercise tolerance experiments [49]. The genotype identification of the AMPKα2^−/−^ mice is shown in the (Supplementary Materials Figure S7A–C). The mice in the ISO group were subcutaneously injected with single ISO doses of 5 mg/kg body weight [16] (corresponding details: the concentration of the ISO solution was 0.5 mg/mL and the injection volume was 0.01 mL/g body weight). The mice in the vehicle group were injected subcutaneously with an equal volume of saline at 10 mL/kg body weight. After 6 weeks of running training according to the previously described exercise protocol, the mice in the running group were stimulated with the same ISO or saline doses as those in the sedentary group. Heart tissue was then harvested from some mice 1 day after the ISO or saline injection to evaluate the expression of inflammatory components and other indicators in vivo (ISO 1-day group), while heart tissue was harvested from the other mice 3 days after the ISO injection to evaluate the myocardial infiltration of inflammatory cells, etc. (ISO 3-day group) (Figure 1A).

### 4.3. Citrate Synthase Activity Assay

The citrate synthase activity in the skeletal muscles of the mice was analyzed using an absorbance-based citrate synthase activity assay (GENMED, Shanghai, China, GMS50130.2). Briefly, gastrocnemius muscle tissue was harvested from the mice, washed quickly in cold, phosphate-buffered saline (PBS), placed in liquid nitrogen and then immediately frozen. Next, the muscle tissue was ground into a powder in liquid nitrogen and was subsequently added to lysate and mixed thoroughly. The homogenate was incubated on ice for 30 min, with powerful vortexing being carried out for 30 s every 10 min during this period. Subsequently, the homogenate was centrifuged at 10,000× *g* for 10 min at 4 °C. The supernatant was extracted and stored at −80 °C prior to further analysis. All examination procedures were conducted in accordance with the manufacturers’ instructions. The absorbance was measured at 412 nm using a Multiskan GO instrument (Thermo Fisher Scientific Cellomics, Pittsburgh, PA, USA). During this period, four measurements were taken every 5 min for 15 min. All concentration values were in the linear range of the standard curve and were calculated based on known protein concentrations. The results showed that citrate synthase activity was significantly higher in the skeletal muscle of mice in the exercise training group (Figure S7D,E).

### 4.4. Isolation and Culture of Primary Neonatal Mouse Cardiomyocytes (NMCMs)

Cardiomyocytes were isolated from 1- to 3-day-old neonatal C57BL/6J mice and cultured as described previously [50]. In brief, the cardiomyocytes were collected using trypsin and collagenase type II (Gibco, Carlsbad, CA, USA). The dissociated cells were then plated on 100 mm culture dishes in Dulbecco’s modified Eagle’s medium (DMEM) with 15% fetal bovine serum and incubated for 2 h. The non-attached cardiomyocyte-rich fraction was plated on plastic dishes (5 × 105 cells/dish) and 100 μmol/L bromodeoxyuridine (Sigma St. Louis, MO, USA) was added to prevent fibroblast proliferation. The cells were incubated in a serum-free medium for 3–4 h prior to drug or mock treatment.

### 4.5. Enzyme-Linked Immunosorbent Assay (ELISA)

The levels of monocyte chemoattractant protein-1 (MCP-1), monocyte chemoattractant protein-5 (MCP-5) and interleukin-18 (IL-18) in the mouse heart tissue were measured using ELISA kits, as described previously. Briefly, heart tissue was harvested from the mice, immediately frozen in liquid nitrogen and then homogenized in a lysis buffer. The procedures were conducted according to the manufacturers’ instructions and the absorbances were measured at 450 nm using a Multiskan GO instrument (Thermo Fisher Scientific Cellomics, Pittsburgh, PA, USA). All concentration values were in the linear range of the standard curve and were calculated based on known protein concentrations.

### 4.6. Measurement of Reactive Oxygen Species (ROS) Levels

The production of reactive oxygen species (ROS) in neonatal mouse cardiomyocytes and the left ventricular myocardium was determined using dihydroethidium (DHE; Invitrogen Molecular Probes, Eugene, OR, USA) staining. Briefly, the transverse cryosections (8 μm thick) of frozen hearts were fixed with acetone for 15 min and washed three times with 0.01% PBS before being incubated with DHE (5 μM) for 30 min at room temperature. The heart sections were stained with 4,6-diamidino-2-phenylindole (DAPI; Wako Pure Chemical Industries Ltd., Osaka, Japan) for the visualization of the nuclei. Images were obtained using a laser scanning confocal microscope (Carl Zeiss Inc., Thornwood, NY, USA), with excitation/emission at 488/555 nm, respectively. To detect the ROS in the NMCMs, serum-starved NMCMs were incubated with 5 mmol/L of DHE for 15 min at 37 °C in 5% CO_2_ and 95% air. The cardiomyocytes were then stained with Hoechst 33342 (Invitrogen Molecular Probes, Eugene, OR, USA) to visualize the nuclei. The fluorescence intensity was measured and analyzed using a Cellomics Array Scan VTI HCS Reader (Thermo Fisher Scientific Cell omics, Pittsburgh, PA, USA) with the Morphology Explorer Bio Application. Cell images were acquired using excitation wavelengths of 386 and 535 nm, with a 300 ms exposure time. The levels of ROS were expressed as the mean fluorescence intensity [51]. The DHE fluorescence was quantified using Image-Pro Plus 6.0 (Media Cybernetics, Bethesda, MD, USA).

### 4.7. Histochemistry

The hearts were harvested from the mice and washed with cold phosphate-buffered saline. To test the immunohistochemistry, the heart sections were incubated with antibodies against the macrophage marker Mac-3 (1:200 dilution; BD Biosciences, San Jose, CA, USA). The sections were imaged using a NanoZoomer-SQ digital slide scanner (Hamamatsu Photonics, Shizuoka, Japan). To evaluate macrophage infiltration, 10 fields were randomly selected from each section and the ratio of the positively stained area to the total myocardial area was calculated using Image-Pro Plus 6.0 (Media Cybernetics, Bethesda, MD, USA).

### 4.8. Western Blotting

Cardiac samples were collected in a lysis buffer (10 mmol·L^−1^ of Tris–HCl, pH 7.4, 100 mmol·L^−1^ of NaCl, 1 mmol·L^−1^ of EDTA, 1 mmol·L^−1^ of EGTA, 1 mmol·L^−1^ of NaF, 20 mmol·L^−1^ of Na4P2O7, 2 mmol·L^−1^ of Na3VO4, 1% Triton X-100, 10% glycerol, 0.1% sodium dodecyl sulfate (SDS), 1% deoxycholic acid, 1 mmol·L^−1^ of PMSF and 1 g·mL^−1^ of aprotinin). The levels of NLRP3, caspase-1, p-AMPK, AMPK and GAPDH were examined using Western blotting. Heart lysate was subjected to sodium dodecyl sulfate-polyacrylamide gel electrophoresis (SDS–PAGE) and blotted on nitrocellulose membranes. Protein samples (40 μg) were separated via electrophoresis on 12% SDS polyacrylamide gels and transferred to polyvinylidene fluoride membranes. The membranes were then incubated with primary antibodies for at least 8 h at 4 °C (NLRP3, 1:500 dilution; p-AMPK, 1:1000 dilution; AMPK, 1:1000 dilution; pro-caspase-1, 1:1000 dilution; caspase-1, 1:1000 dilution; GAPDH, 1:10,000 dilution). After the membranes had been incubated with the corresponding HRP-conjugated secondary antibodies (ZSGB-BIO, Beijing, China), the protein bands were visualized using Immobilon Western Chemiluminescent HRP Substrate (Millipore Corporation, St. Burlington, MA, USA). Blotting images were obtained using a Syngene Gene Gnome-XRQ-NPC imager (Syngene Company, Cambridge, UK), and the proteins were quantified by calculating the grayscale value of each band using ImageJ 1.52 (Media Cybernetics, MD, USA).

### 4.9. Immunofluorescence

For the immunofluorescence staining, serial transverse cryosections (8 μm thick) of frozen hearts were cut using a microtome (Leica, Wetzlar, Germany) and placed on polylysine-coated glass slides. The slices were then incubated with the primary antibodies against the endothelial cell marker CD31 (1:100 dilution; Cell Signaling Technology, #3528, Danvers, MA, USA) and intercellular adhesion molecule 1 (ICAM-1; 1:100 dilution; Abcam, #ab282575, Cambridge, MA, USA), followed by incubation with fluorescence-conjugated secondary antibodies. The sections were also stained with 4′,6-diamidino-2-phenylindole (DAPI; Wako Pure Chemical Industries Ltd., Osaka, Japan) for the visualization of the nuclei.

### 4.10. Statistical Analysis

The data were expressed as the means ± SEMs. All samples were independent. For the parametric analysis, a one-way ANOVA with a two-sided Tukey’s post-hoc test was used to analyze the differences between groups when the data followed a normal distribution and had equal variances. For the nonparametric analysis, a Kruskal–Wallis ANOVA combined with a two-sided Dunn’s multiple comparison post-hoc test was performed when more than two groups were evaluated. The data were analyzed using GraphPad Prism software (version 8.4; GraphPad Software Inc., San Diego, CA, USA) and SPSS 22 (IBM Co., Armonk, NY, USA). A value of *p* < 0.05 was considered to indicate statistical significance.

## 5. Conclusions

In conclusion, exercise training inhibited the ISO-induced activation of NLRP3 inflammasomes and cardiac inflammation in mice. Exercise training exerted cardioprotective effects by activating AMPK, which inhibited NLRP3 inflammasome activation. Our study showed that exercise training could prevent sympathetic stress-induced cardiac inflammatory injuries and identified a novel mechanism for the cardioprotective effects of exercise.

## Data Availability

Data is contained within the article or supplementary material.

## Supplementary Materials

The following supporting information can be downloaded at: https://www.mdpi.com/article/10.3390/ijms24119263/s1.
