# Supplementary material for: Exercise Training Attenuates Acute β-Adrenergic Receptor Activation-Induced Cardiac Inflammation via the Activation of AMP-Activated Protein Kinase
Source: Int J Mol Sci. 2023 May 25;24(11):9263. doi: 10.3390/ijms24119263 (PMC10252890; doi:10.3390/ijms24119263)
Supplement: Supplementary file 1 [file ijms-24-09263-s001.zip › ijms-2355707-supplementary.pdf]

Supplementary materials

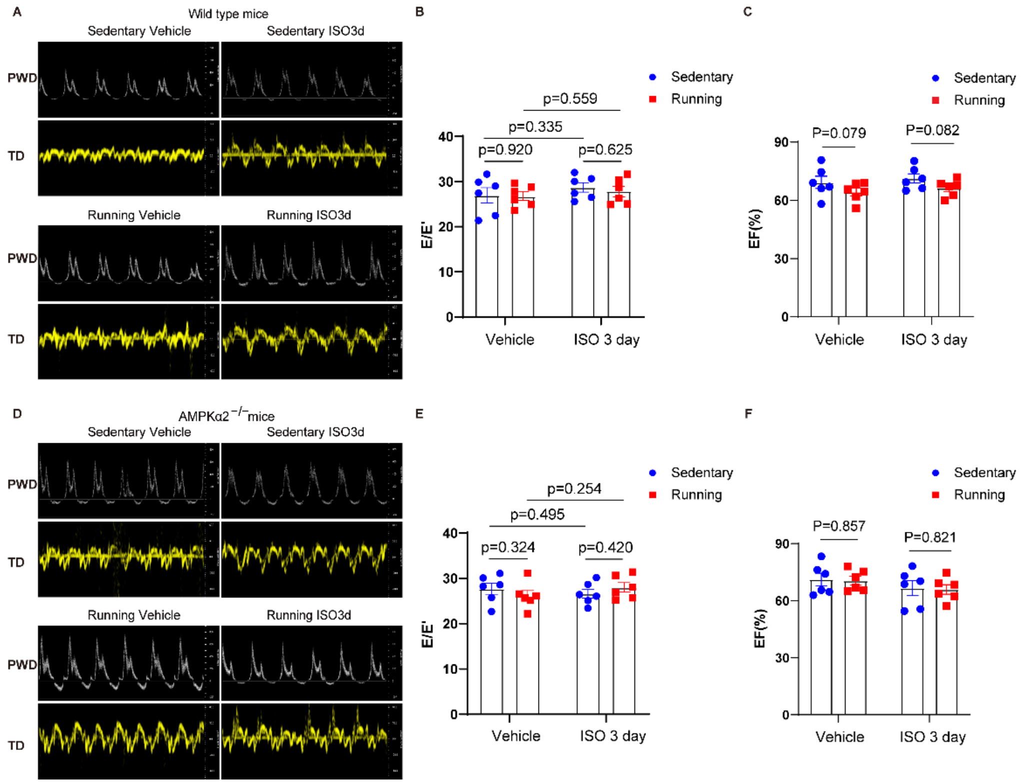

Figure S1 : At 3 days after ISO insult, there were no alterations in the diastolic and systolic function of the mice hearts: (**A**) representative images of the pulsed wave Doppler (PWD) analysis across the mitral flows and the tissue Doppler (TD) analysis of the mitral valve rings in the different groups of WT mice; (**B**) a statistical graph of the E/E′ ratio in the WT mice; (**C**) a statistical graph of the ejection fraction (EF) in the wild-type mice. (**D**) representative images of the pulsed wave Doppler (PWD) analysis across the mitral flows and the tissue Doppler (TD) analysis of the mitral valve rings in the different groups of AMPK$\alpha 2^{-/-}$ mice; (**E**) a statistical graph of the E/E′ ratio in the AMPK$\alpha 2^{-/-}$ mice. (**F**) a statistical graph of the ejection fraction (EF) in the AMPK$\alpha 2^{-/-}$ mice. Note: ISO, isoprenaline; Running, exercise training; n = 6. The data are the mean ± SEM from a one-way ANOVA with a Tukey's post-hoc test.

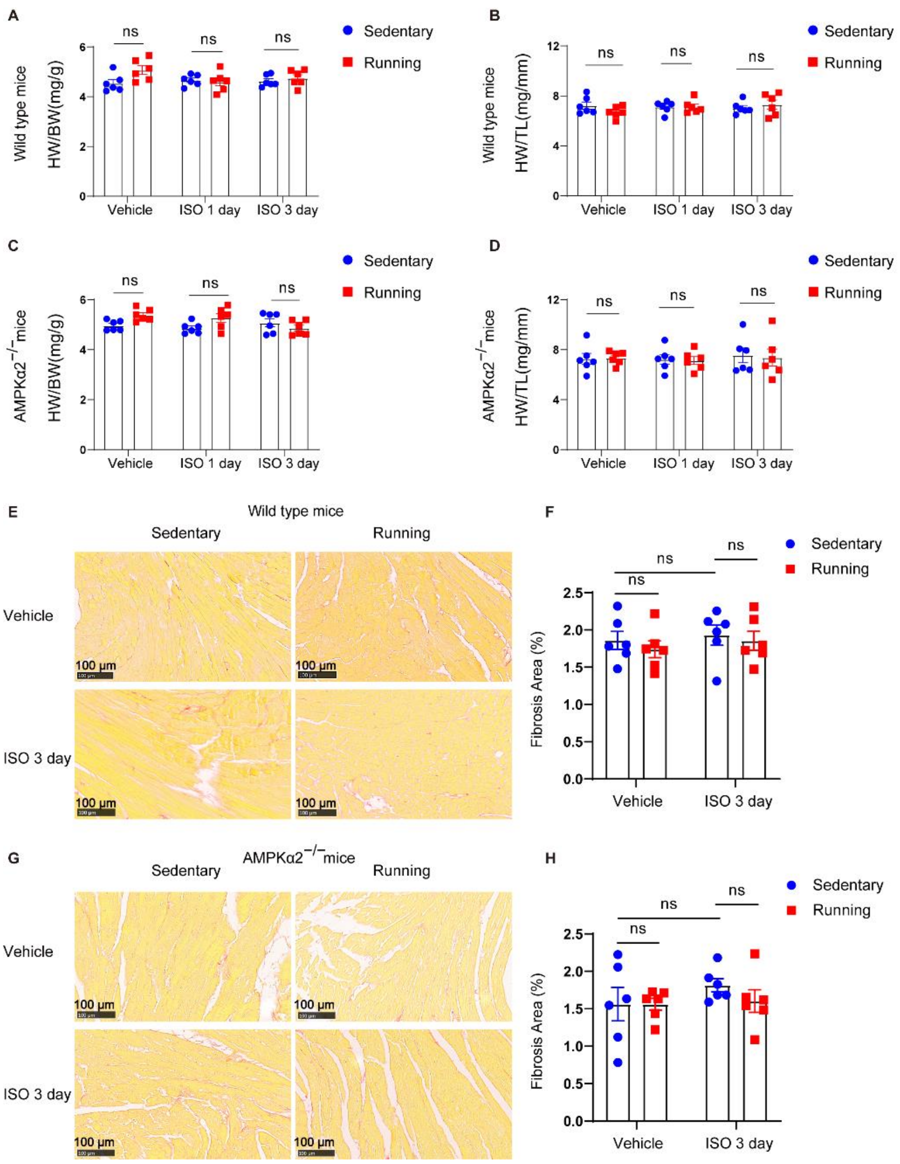

Figure S2: At 3 days after ISO stimulation, neither wild-type mice nor AMPKα2$^{-/-}$ mice had hypertrophy and fibrosis in their heart tissue: (**A,B**) Heart weight-to-body weight ratio (HW/BW) and body weight-to-tibial length ratio (BW/TL) in wild-type mice; (**C,D**) Heart weight-to-body weight ratio (HW/BW) and body weight-to-tibial length ratio (BW/TL) in AMPKα2$^{-/-}$ mice; (**E,F**) Representative diagram and statistics of Sirius scarlet staining of heart tissue in wild-type mice (bar = 100 μm); (**G,H**) Representative diagram and statistics of Sirius scarlet staining of heart tissue in AMPKα2$^{-/-}$ mice (bar = 100 μm). ISO, isoprenaline; Running, exercise training; HW, heart weight; BW, body weight; TL, tibia length. n = 6. Data are mean ± SEM; one-way ANOVA with Tukey's post-hoc test or Kruskal–Wallis ANOVA with post-hoc Dunn’s multiple comparison tests were used.

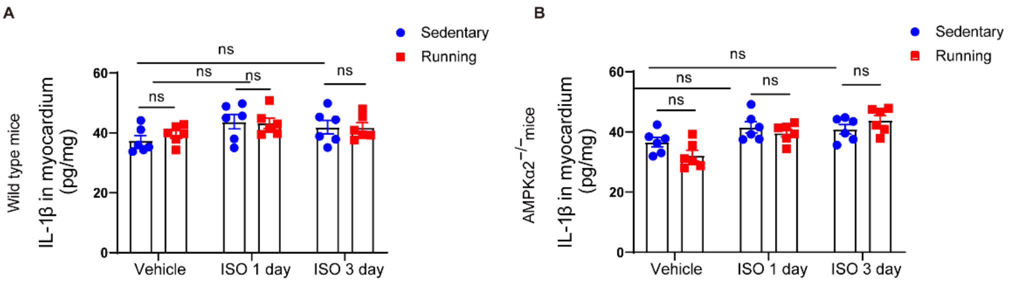

Figure S3: IL-1β did not change significantly in the hearts of mice 1 or 3 days after ISO stimulation. Detection of IL-1β in mouse heart tissue after exercise training and ISO stimulation: (A) Content of IL-1β in the heart tissue of wild-type mice; (B) Content of IL-1β in the heart tissue of AMPKα2$^{-/-}$ mice. ISO, isoprenaline. n = 6. Data are mean ± SEM; one-way ANOVA with Tukey's post-hoc test or Kruskal–Wallis ANOVA with post-hoc Dunn's multiple comparison tests were used.

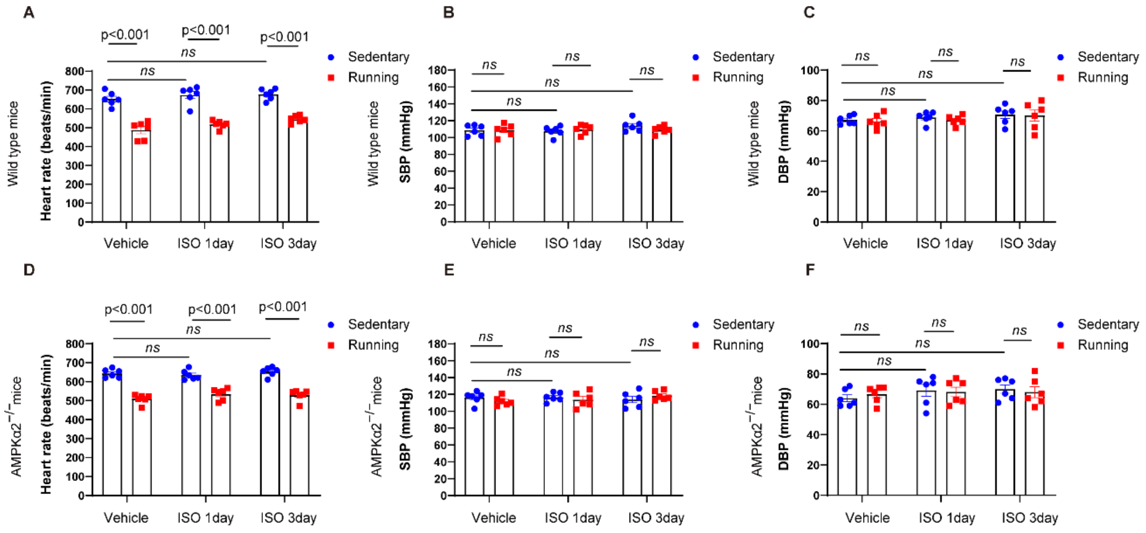

Figure S4: In this study, 6 weeks of exercise training reduced heart rates, but not blood pressure, among the mice: (**A**) the heart rates of the WT mice in the exercise training group compared to those in the sedentary group; (**B-C**) the systolic and diastolic blood pressure in the sedentary and exercise training groups of WT mice; (**D-F**) the heart rates and blood pressure of the AMPKα2$^{-/-}$ mice in the exercise group. Note: ISO, isoprenaline; Running, exercise training; SBP, systolic blood pressure; DBP, diastolic blood pressure; n = 6. The data are the mean ± SEM from a Kruskal–Wallis ANOVA with a post-hoc Dunn's multiple comparison test.

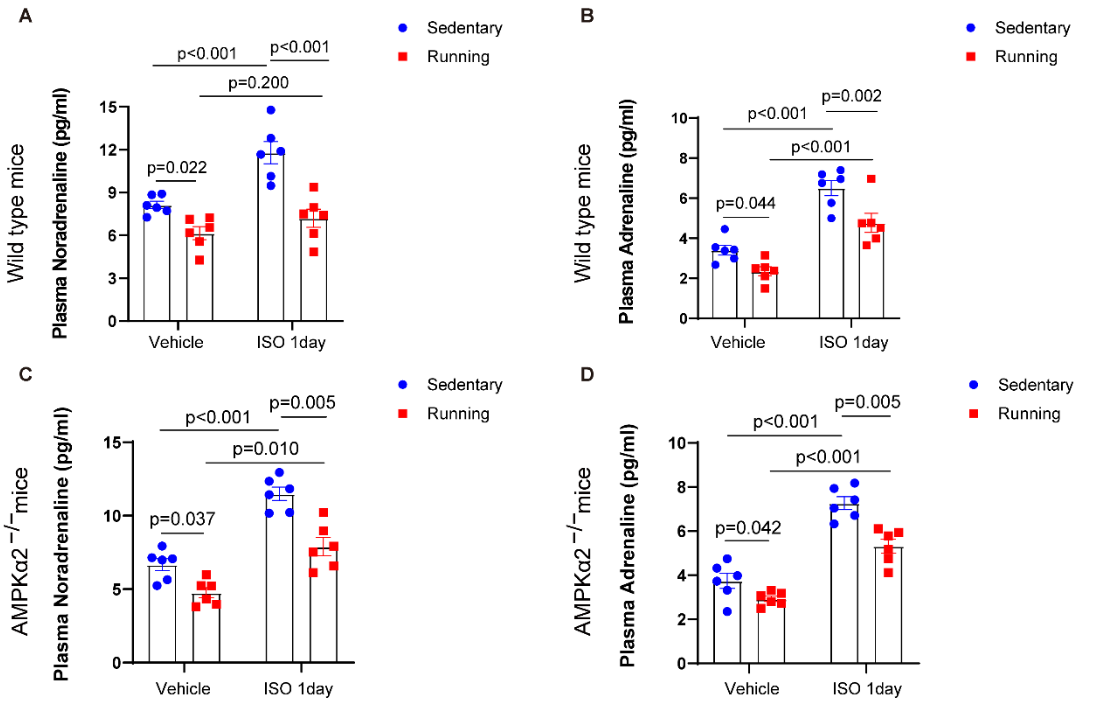

Figure S5: Exercise training was able to decrease plasma catecholamine levels in the WT and AMPKα2$^{-/-}$ mice: (**A-B**) the concentrations of (**A**) noradrenaline and (**B**) adrenaline in the plasma of the WT mice, as determined by an ELISA; (**C-D**) the concentrations of (**C**) noradrenaline and (**D**) adrenaline in the plasma of the AMPKα2$^{-/-}$ mice, as determined by an ELISA. Note: ISO, isoprenaline; Running, exercise training; n = 6. The data are the mean ± SEM from a one-way ANOVA with a Tukey's post-hoc test.

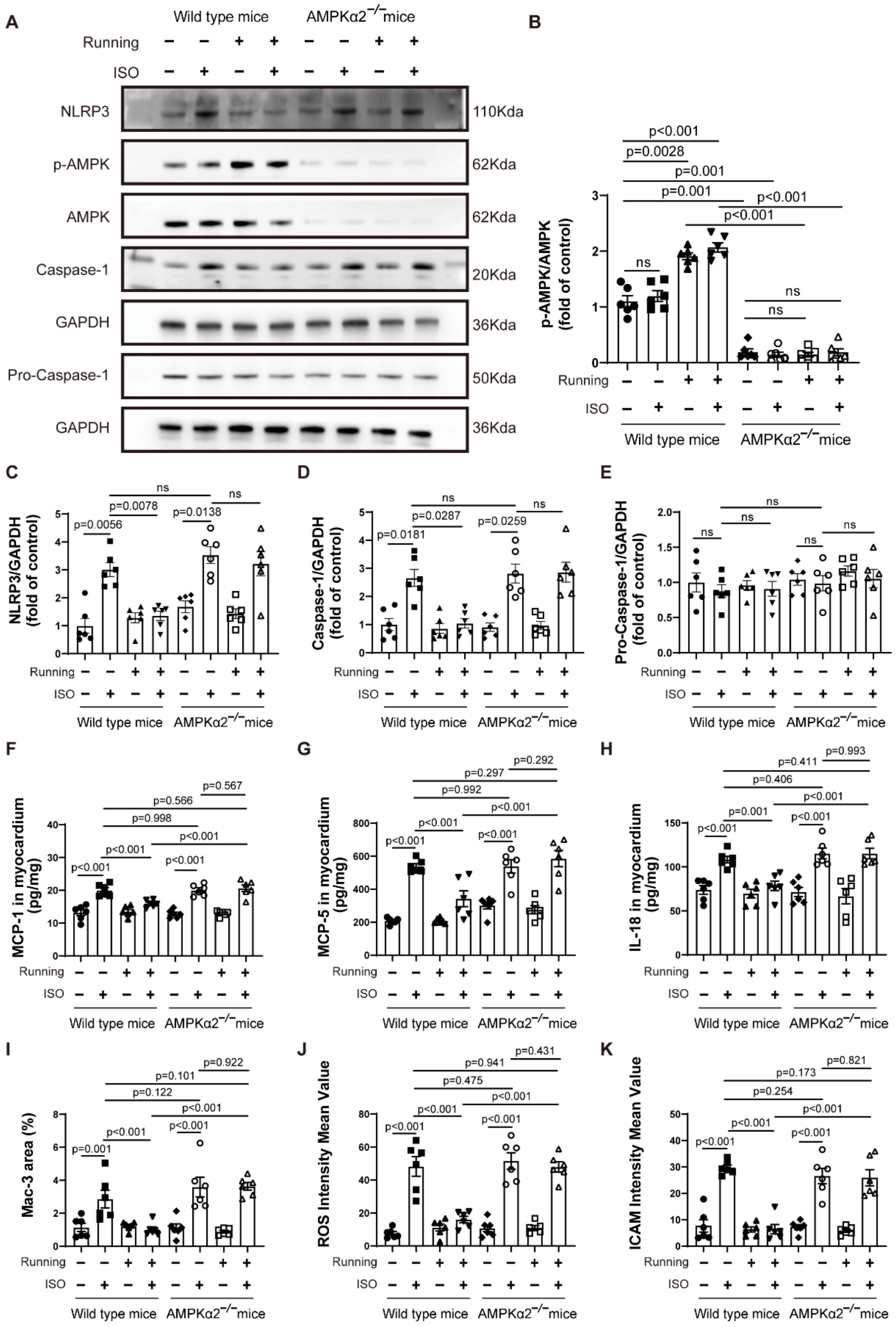

Figure S6 : Exercise training alleviated cardiac inflammation in the WT mice but not in the AMPK$\alpha 2^{-/-}$ mice: (**A**) the Western blot analysis of NLRP3, p-AMPK, AMPK, pro-caspase-1 and caspase-1 levels in the heart tissue of the WT and AMPK$\alpha 2^{-/-}$ mice; (**B–E**) the quantitative analysis of the relative protein expression of (**B**) p-AMPK, (**C**) NLRP3, (**D**) caspase-1 and (**E**) pro-caspase-1 in the heart tissue; (**F-H**) the concentrations of (**F**) MCP-1, (**G**) MCP-5 and (**H**) IL-18 in the myocardium of the mice, which were

determined by an ELISA; (**I**) representative immunostaining and quantification of Mac-3 (macrophage marker) in the heart at 3 days after ISO treatment; (**J, K**) the quantification of the fluorescence intensity of the ROS and ICAM-1 in the mouse heart sections. Note: ISO, isoprenaline; Running, exercise training; MCP-1, monocyte chemoattractant protein-1; MCP-5, monocyte chemoattractant protein-5; ICAM-1, intercellular adhesion molecule 1; n = 6. The data are the mean ± SEM from a Kruskal–Wallis ANOVA with a post-hoc Dunn's multiple comparison test.

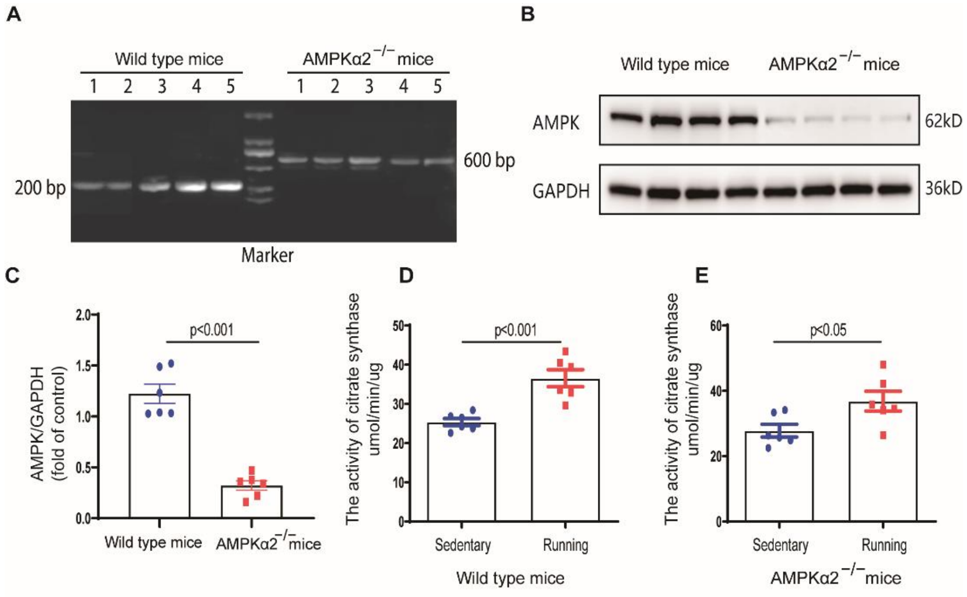

Figure S7: A validation of the effectiveness of AMPK knockout and exercise training: (**A**) the genotyping results (600bp bands detected in the AMPK$\alpha 2^{-/-}$ mice and 200bp bands detected in the wild-type (WT) mice); (**B-C**) the protein level verification of the AMPKα2 knockout success; (**D-E**) the gastrocnemius skeletal muscle citrate synthase activity in the mice, which was significantly increased after exercise compared to that in the sedentary group. Note: Running, exercise training.; n = 6. The data are the mean ± SEM from a one-way ANOVA with a Tukey's post-hoc test.
